# Negotiating precarity – employment experiences of Canadian young adults in social housing

**DOI:** 10.1177/10519815261432596

**Published:** 2026-03-23

**Authors:** Julia Jansen-van Vuuren, Terry Krupa, Rosemary Lysaght, Carrie A Marshall, Daniella Aguilar, Fiona Drake, Nicole Bobbette

**Affiliations:** 1 School of Rehabilitation Therapy, Queen’s University, Kingston, Ontario, Canada; 2 School of Occupational Therapy, Western University, London, Ontario, Canada; 3 Kingston & Frontenac Housing Corporation, Kingston, Ontario, Canada

**Keywords:** environmental factors, informal employment, mental health, quality of life, unemployment, occupation

## Abstract

**Background:**

Young adults living in social housing frequently face the double precarity of vulnerable housing and unstable employment, negatively affecting their health and well-being.

**Objective:**

This study explored the experiences of Canadian young adult social housing residents to better understand their employment needs, challenges, and goals.

**Methods:**

Thirteen young adults residing in social housing in a mid-sized Canadian city were interviewed using an online platform. Interviews were transcribed verbatim and analysed based on Braun and Clark's inductive thematic analysis.

**Results:**

Although participants valued the concept of work, only two were formally employed at the time of interview. Three broad narratives related to employment were identified: the ‘persistently unemployed’, ‘single mothers’, and ‘youth’. Themes cutting across these narratives were developed related to work as a precarious experience and provide insight into how social housing and available supports influenced participants’ lived experience of employment.

**Conclusions:**

Findings highlight the complex relationship between employment, housing, and well-being. Young adults living in social housing were not a homogenous group with respect to employment, yet the shared context in which they live contributed to shaping their employment experiences, priorities, and goals. A Capabilities lens was applied to consider what opportunities and freedoms social housing residents have or need to experience the well-being and inclusion benefits of employment and to promote individual and community flourishing.

## Introduction

Secure, quality housing is considered a social determinant of health affecting overall well-being.^1,2^ Social housing provides affordable, secure accommodation for marginalised populations; however, evidence indicates low employment rates among residents,^3–5^ limiting access to the well-being benefits typically associated with work. This inequity mirrors patterns seen in other low-income populations.^4,6–10^

There are compelling reasons to foster higher employment among social housing residents. Beyond the financial benefits, employment can provide structure and routine, strengthen social connections and responsibilities, and develop personal skills.^11–14^ Conversely, unemployment and precarious employment—characterised by insecurity, instability, limited rights and benefits, vulnerability, and inadequate pay—are linked to significant, long-term negative effects on mental and physical health, earnings, future employment opportunities, social participation, and overall well-being.^15–19^ Of particular interest in this study are young adults living in social housing, as this life stage is critical for developing employment and life skills and entering the workforce. Understanding their employment needs, challenges, and goals is essential for supporting well-being and enabling community contribution and success.

### Social housing

Social housing is known by various terms, including council, subsidised, project, or public housing, which differ across countries and jurisdictions.^20^ In this study, ‘social housing’ denotes residential accommodation with below-market rents distributed by government or non-profit landlords according to eligibility criteria.^21^ Despite some countries (e.g., Canada) legislating the right to housing, this right is not always protected, and many people remain in vulnerable housing situations, particularly amid rising costs.^22^

Some social housing residents have previously experienced homelessness or cannot afford market rents or sustain home ownership due to relationship breakdown, domestic violence, eviction, job loss, or poor health.^5,23,24^ In some contexts, a substantial proportion of residents are refugees or migrants who face intersectional housing challenges, including racism and discrimination, low vacancy rates, overcrowding in larger families, and minimal resources and social connections.^25,26^ Others have grown up in social housing and remain with family members or move into their own apartment, often after having a child.^5^

### Employment and social housing

Employment—occupation involving the formal exchange of labour for income—is typically seen as a means to enhance well-being and mitigate precarity. However, unemployment disproportionately affects persons who are already disadvantaged, and social housing residents often face the double precarity of insecure housing and unstable employment or weak employment histories.^27–29^ Many have family responsibilities or are single parents, with childcare frequently inadequate or unaffordable.^12,30,31^ Physical and mental health conditions and disability also present significant employment barriers.^5,9,30^ Attitudinal barriers, including societal perceptions of social housing, can restrict employment opportunities^4,11,32^; however, Haley^33^ found that among young adults, health, education, and the number of adults in the household were stronger predictors of labour-force participation than stigma. Limited education and skill development,^30,31^ as well as mismatches between skills and available jobs,^4^ further constrain employment.

Tenants who are employed are often in casual/part-time, low-paid work that is temporary, insecure, and inadequate for self-sufficiency.^4,11,32^ Residents may be reluctant to pursue employment if they perceive limited financial gain and risk of losing benefits such as rental subsidies.^34^ Neighbourhood-level economic disadvantage and household deprivation are associated with persistent unemployment,^35^ while locally concentrated social ties in deprived neighbourhoods can also prolong unemployment.^36^ A recent scoping review found that social housing residents face multiple, complex personal and environmental barriers to work, and most existing programs are broad self-sufficiency initiatives rather than targeted employment supports.^37^ The review also identified a lack of qualitative research on the employment experiences of young adults in social housing.^37^

### Conceptual framework

This research draws on the Capabilities Approach,^38^ a multidisciplinary social justice framework that conceptualises capabilities as the ‘substantive freedoms’ people have to live the lives they have ‘reason to value’.^39 p87^ Adequate housing and employment are recognised as central capabilities for living with dignity in a socially just society.^40^ Housing can also enable other capabilities^41^:

…[Housing] enables us to increase our capabilities by allowing us to rest; offering us somewhere to store our belongings and to clean ourselves; providing a space for personal and social relations, a space to foment creativity, a point of reference, a workplace or leisure space; and, as a symbol of belonging to a community, enabling our political participation. Housing therefore gives us the ability to achieve the functionings or states of well-being that we can understand as a “home”. ^42 p193^

The Capabilities framework can be applied to specific populations and contexts to examine the extent to which individuals have the substantive freedoms to pursue valued opportunities, and to identify personal, social, and environmental conversion factors that enable or constrain these capabilities. Exploring young adult Canadian social housing tenants’ lived experiences of employment—including strengths, barriers, and aspirations—is important for addressing inequities.

## Methods

### Data collection

The researchers used a qualitative exploratory design to understand the employment experiences, goals, and barriers of young adults aged 18–34 living in social housing. Ethical clearance was obtained from the university ethics board. Inclusion criteria were informed by Statistics Canada's first two demographic classifications for adults, ages 15–34. The lower age limit was set at 18, when individuals gain full legal rights and responsibilities, and the 25–34 category was included to reflect the extended process of achieving personal and financial independence in the current climate^43^ and to broaden the study scope.

We recruited a convenience sample of young adults in the local social housing corporation in a mid-sized city in Ontario, Canada, with assistance from the corporation's support manager. This site was selected because it is the city's largest social housing provider and an existing partnership was already established. Recruitment flyers were distributed to letterboxes and posted in common areas; individuals scanned a QR code to email the researchers and express interest. Follow-up emails included a letter of information and consent outlining study aims, risks and benefits, voluntary participation, confidentiality, and the right to withdraw at any time without consequence. For those who agreed, a mutually convenient interview time was scheduled, and verbal consent was confirmed before starting the interview. Snowball sampling was also later employed. Participants were demographically diverse in gender, age, city region, and length of time in social housing. All interviews except one were conducted via Zoom, and participants received $20 CAD in appreciation of their time.

### Data analysis

Audio-recorded interviews were transcribed and analysed using reflexive thematic analysis.^44^ This analytic framework aligns with a qualitative exploratory approach by recognising multiple realities, contextual meaning, and researcher subjectivity as a resource.^44^ Transcripts were analysed inductively and iteratively, with attention to potential assumptions and prioritising participants’ voices. The authors familiarised themselves with the data through repeated reading of transcripts, before uploading files to NVivo for data management. Initial codes were generated through open coding across the dataset to label and categorise text segments, followed by focused coding to condense related concepts. Two authors independently reviewed two transcripts, identifying key themes (patterns of meaning) that captured each participant's main narrative before meeting to discuss. This process continued for subsequent transcripts. Themes were developed into consistent, coherent accounts with detailed written analyses supported by participant quotes with pseudonyms. For the Discussion, the authors examined connections between themes and interpreted meanings in relation to the Capabilities Approach and the social housing context to deepen critical analysis of the data.^45^

### Reflexivity statement

Recognising researcher positionality, assumptions, and potential biases is critical for transparency and rigour in qualitative research. In this study, the researchers were primarily from occupational therapy or rehabilitation backgrounds and brought varied research and professional experience, including in mental health, homelessness, and community participation, inclusion, and well-being. One author, the housing corporation support manager, has extensive experience with the population and has contributed to previous research on supporting well-being for social housing residents. She assisted with recruitment flyer distribution and provided ongoing consultation on contextual influences and the housing corporation's specific challenges and strengths. However, to minimise conflict-of-interest and protect participant confidentiality, she had no involvement in interviewing or access to transcripts, remaining blinded to participants’ identities. All Zoom interviews were conducted by a postdoctoral fellow experienced in qualitative interviewing to ensure consistency.

## Findings

### Sample description

#### Demographic characteristics

Thirteen young adult social housing residents participated in the study; Table 1 presents their key demographic characteristics. Despite only two participants being formally employed at the time of interview, all engaged in various occupations, including education (e.g., completing high-school subjects), searching and applying for work, informal work, caring for children/family/pets, and daily life routines encompassing self-care and domestic tasks. Several participants indicated they had minimal structure or routine, describing ‘relaxing at home’, playing computer games, or occasionally socialising with friends and family as favoured activities.

**Table 1.** Demographic characteristics of study participants.

Characteristics	n = 13 (%)
Age (years)	
18–24	5 (38.5)
25–29	2 (15.4)
30–34	6 (46.2)
Gender	
Female	7 (53.8)
Male	6 (46.2)
Highest level of education	
<Grade 12	3 (23.1)
Grade 12	5 (38.5)
Incomplete college	3 (23.1)
Certificate	1 (7.7)
Diploma	1 (7.7)
Current employment status	
Unemployed	11 (84.6)
Some formal employment	2 (15.4)
Government support	
ODSP	6 (46.2)
OW	4 (30.8)
None	3 (23.1)
Approximate time living in social housing as an adult (years)	
<1	3 (23.1)
1–2	6 (46.2)
3–4	2 (15.4)
5–6	0
7–8	1 (7.7)
9+	1 (7.7)
Main reported health issue	
Anxiety and/or depression	6 (46.2)
Schizophrenia	1 (7.7)
ASD	1 (7.7)
Epilepsy	1 (7.7)
Dyslexia	1 (7.7)
None reported	3 (23.1)

Note: ODSP = Ontario Disability Support Program; OW = Ontario Works (i.e., financial support based on need); ASD = Autism Spectrum Disorder

#### Participant employment narratives

During theme development, our analysis revealed three participant sub-groups, with distinct narratives and shared characteristics, priorities and work-related goals, including future employment expectations. Hence, we constructed three broad categories: ‘persistently unemployed’ (unemployed for four or more years), ‘single mothers’ (women living alone with one or more children), and ‘youth’ (participants under 25 at an early stage of labour market entry). These categories were not mutually exclusive and served as interpretive tools, not rigid typologies. For example, some ‘single mothers’ were also under 25 or ‘persistently unemployed’; however, their identity as mothers distinguished them and most strongly shaped their employment priorities and values. Each group comprised approximately one third of the sample.

##### ‘Persistently Unemployed’ – Employment is not feasible for me

For these participants, sustaining formal employment was viewed – by themselves and others – as unlikely. They were older young adults (aged 27–34) with limited work experience, weak educational backgrounds, and fewer resources to pursue employment beyond entry-level work. Participants reported significant health challenges, including autism spectrum disorder, depression and anxiety, and epilepsy, and were all Ontario Disability Support Program (ODSP) recipients. Ongoing unemployment appeared to compound inertia and reduce ability to consider work as a viable future goal. As one participant suggested, his extended employment absence impacted his identity as a worker, compromising employer perceptions:

I wonder, like after it's been, I want to say at least a good 5 years since I've worked, what would I tell somebody going to apply to a job, like to go back, when they ask for my resume… And I give them something that's been so long back, and they're like, ‘Well, why has it been so long since you worked?’ (*James*).

Despite sensing that entering the workforce was unlikely, the concept of employment still appealed to these participants. One participant expressed how she missed the routine and purpose of work, in contrast to the current lack of structure described by many:

I sleep when I sleep, and I'm awake when I'm awake… it's kind of how the cookie crumbles… But I liked the schedule of [work], and I like getting up and going to work and being useful and stuff like that, whereas now it's just kind of… Do I sleep? Do I play video games? … I don't know what to do. (*Chloe*).

All could describe their dream job even if they had no clear pathway to achieve it. One participant loved animals and wanted to become a veterinary technician but was still finishing her high-school diploma. Another liked the idea of being a pilot but acknowledged this was unlikely.

##### ‘Single Mothers’ – Employment as contributing to a better life for my children

Four ‘single mothers’, with children ranging from babies to pre-teens, organised their lives around parenting. As one stated: ‘A normal day for me now, because I’m not working – so, I’m just taking care of my son… just get him ready for school, when he gets off the bus like dinner, bath, homework, bed.’ (*Mia*). Apart from one employed ‘single mother’, these participants received government unemployment benefits, and one was applying for ODSP.

All the mothers sought a better future for their children and considered work important in role modeling and instilling a sense of potential in their children. One mother expressed it in these terms:

Just because we're in a low-income situation doesn't mean that we're bad people. Like it's just we have… barriers. There's so many times I say to my daughter, you can do anything when you grow up. Like you can do anything, you just have to work hard. Because unfortunately, you stop focusing on the work, that's when everything else can take over, and that's what happened with me. And I loved working. (*Dakota*).

Another mother who was currently working part-time in retail aspired to a different life, having completed a law enforcement-related diploma:

It was really motivating for me to want to do different, and instead of taking the other route that most people take when they're given the life I have, I just decided to do something else… I feel like [a career in law enforcement] would be something positive in my community. (*Isla*).

##### ‘Youth’ – Aspiring to a future career

The ‘youth’ were under 25 and two had recently finished high-school. One participant was working part-time for pay, while the others were more recently unemployed and two received government benefits. However, all of them conceptualised a hopeful future in a career of personal interest, even if not currently searching for employment and focusing instead on stabilising their mental health or studying. As one stated, ‘Right now, I'm not looking for work. … I want to focus all my time on school. And once I get that I might look for a job.’ (*Nick*).

Most ‘youth’ seemed conscious of the stigma associated with social housing and welfare, motivating them to get off government assistance. All wanted meaningful and fulfilling future jobs with some autonomy, as expressed by one participant:’… something where I could just be waiting most of the time for work to do, for some exciting work to do, that's going to provide me with some kind of a challenge as well. I like problem solving.’ (*Ethan*).

### Thematic analysis: Factors shaping employability

**Figure 1.**
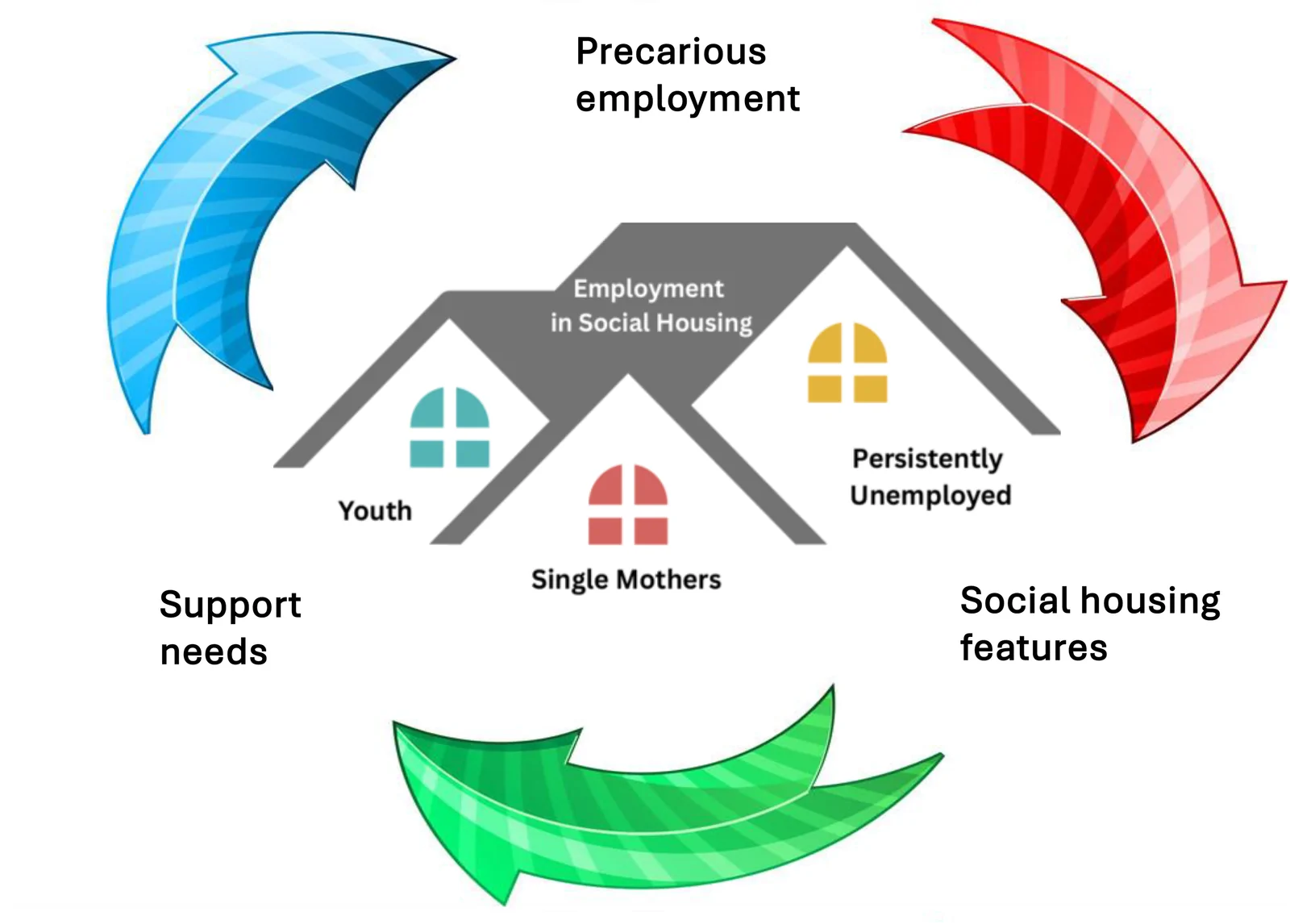
Visual representation of themes.

The following section develops themes that crosscut the three employment narratives. Figure 1 provides a visual representation of themes depicting how young adult social housing residents are influenced by their experiences of precarious work, features of the social housing context, and support needs, which in turn are dynamic and interact with each other.

#### Working as a precarious experience

Every participant had experienced short-term, minimum wage, low-skilled jobs, such as in food service, cleaning, and retail – ‘a boring wage slave job…’ (*Lucas*). Participants recounted multiple experiences of being laid off or fired, often for reasons outside of their control, such as businesses changing ownership. Others had made mistakes (e.g., forgetting to lock up after a shift) or missed shifts due to family responsibilities. One ‘single mother’ had finally secured regular work shifts to qualify for childcare, only to lose her job during a family emergency: ‘I had a gut feeling things were going bad in my relationship, and I didn't put work first, and I didn't go to work. And then I ended up lying to the boss and getting fired.’ (*Dakota*).

Participants described unsupportive, inflexible and even discriminatory workplaces e.g., ‘A workplace not pleasant is being yelled at, being told you're useless, you're no good for nothing, you'll never get a job if you can't manage this. It's a simple damn task.… they would talk down to me. Big time.’ (*Alyssa*). One ‘single mother’ perceived workplace racial discrimination and power imbalances, but saw no point in reporting to unsupportive supervisors: ‘I'm the only African American on the team… I'm not gonna try and base it off my skin colour or anything like that, but I do get treated different, as in like they won't give me hours like everybody else.’ (*Isla*). Similarly, a young man who reported a diagnosis of schizophrenia felt exploited by his previous workplace: ‘A lot of the employers in [town] are really cheap. They don't want to pay; they want you to work your guts out and work like a slave. Especially if you have a mental illness, they take advantage of you.’ (*Mark*).

Mental health issues were a major work barrier for many participants; they experienced them as affecting their ability to maintain a schedule and perform adequately in their role, believing employers perceived them as unreliable.

I found out that I had anxiety and depression, and I was having a hard time with working and keeping a job, because I’d often find times where I’d like get days where I just couldn't do anything. So, I’d call into work and make an excuse not to show up, and over time that would happen periodically, and that would affect my job. (*James*).

Another ‘persistently unemployed’ participant was self-medicating with alcohol, which she felt precluded formal employment: ‘To be able to responsibly drink on the job, which is not legal unfortunately… if I could find a job where I could do that… just so I can function, then I wouldn't have a problem…’ (*Chloe*). An unemployed ‘single mother’ of two expressed frustration that her mental health hindered her ability to be a good employee:

I would love to be able to not just have a job, but to be good at it, to be there on time every day and not have the worry of, ‘What if I screw up? What if I can't control myself?’… If I could just fix my brain, and if I could get rose-coloured glasses on my brain so that everything worked the way that it was supposed to, I could do better for my kids, do better for me… we’d all be happier. (*Dakota*).

Other participants expressed a need to stabilise mentally before pursuing formal work, as this ‘youth’ explained:

I lost my last job because I wasn't able to take care of myself and maintain my state of mind well enough, so I feel like I need to get into a spot where I'm better able to do that first, before I actually try and return to the workforce. (*Ethan*).

Several ‘persistently unemployed’ participants described how reliance on a disability pension contributed to experiences of stigma and job discrimination e.g., ‘…as soon as [prospective employers] see ODSP, that I'm on ODSP, they see it as a red flag.’ (*Cameron*). Conversely, an unemployed ‘single mother’ wanted to secure a pension first before seeking work: ‘My goals are just to wait, just hear back from ODSP to see. And I feel that I can work while I'm on ODSP, so after I get that information, then I would like to start looking for work.’ (*Mia*).

Participants shared various other barriers to employment. ‘Single mothers’ specifically, described childcare as a significant challenge: ‘Daycare is not an option here… it's impossible to get in… I applied when [my daughter] was one, and they said in the summer of 2024. Well, that's useless to me, because she'll be in school by then.’ (*Isla*). Others spoke about the challenges of maintaining the right look for employment, as one ‘youth’ stated: ‘Appearances are a really big thing, I’d say… I mean, it's human nature to judge, right?’ (*Lucas*). Although one ‘youth’ had been accepted to university, hoping for a career as a pilot or engineer, others lacked a firm plan for reaching their employment goals. They described work-related behaviours that could be problematic in sustaining future employment, such as job commitment: ‘I'll get three days in and then just give up. … there isn't much that can ever make me do anything that I don't feel like doing.’ (*Ethan*). Another ‘youth’ said he felt ashamed for quitting a job after several days, and fear of failure appeared to constrain his future employment goals: ‘I don't want to have my hopes too high for [computer programming]. Because if I get my hopes really high for that, and I fail, I feel like I'd be really let down.’ (*Nick*).

#### Social housing features impacting employability

Participants spoke about social housing as providing a secure base, including as a foundation for finding and maintaining work. One ‘youth’ shared the importance of housing to shower and present oneself appropriately for work. Prior to entering social housing, participants described transient, challenging or chaotic living situations, which impeded job acquisition. Several participants had been at risk of homelessness: ‘I would not be living like I am today if it wasn't for having this youth housing, I would literally be homeless. So, it literally kind of saved me.’ (*Lucy*). Overall, the ‘youth’ described positive experiences of social housing, perhaps because they were living in newer apartments developed specifically for young people. Considering the current housing crisis, exorbitant commercial rents, long waitlists for social housing, and lack of financial stability, many participants recognised they were fortunate to have social housing, even if they had negative experiences. As one ‘single mother’ expressed: ‘I just broke up with my boyfriend, so life was kind of crazy, not going very well, and then I was a single mum, so I couldn't afford to live, I applied for [social housing] because it was expensive to live elsewhere.’ (*Natalie*).

Despite expressing gratitude for affordable housing, some participants, especially the ‘persistently unemployed’, described how living in social housing negatively affected employment as well as health and safety.

It's probably one of the worst buildings in [town]. Well, there's cockroaches for one. People have problems with bed bugs… We have problems with the homeless in town, and the drug addicts that like to hang out in our stairwell, use them as a washroom, have fires in them, whatever else they decide to do like this. There's people that fight and argue in the hallways. They try and break into the building to get in from the cold and like, it's just… a sad building to be in. (*James*).

Other participants also described pest problems and one ‘persistently unemployed’ participant shared how an unhealthy home environment could transfer to a workplace: ‘I don't want to take cockroaches into a restaurant [workplace]. They stick to your clothes and stuff and then I go to a restaurant and voila! Now, that restaurant has cockroaches.’ (*Alyssa*). The same participant perceived stigma related to living in social housing: ‘I've been told my building is a trap house. It's for druggies and prostitutes.’ (*Alyssa*). Drug use was cited as an issue in social housing by various participants; one participant thought this was a major barrier to work: ‘…you want to know what the people's problem is in [social] housing? Is because they do drugs… stay clean, stay focused and you can get a job… If you're in [social] housing, you have no excuses.’ (*Mark*).

For ‘single mothers’ in particular, the housing environment compromised their sense of the lives their children should live, now and in the future: ‘I don't want this to be all my kids know, because this isn't normal, the way that people just accept this…’ (*Dakota*). To protect their children, they minimised contact with others. One mother described the importance of changing her housing situation if the future she desired for her child was to be realised:

Definitely out of [social housing], that's for sure. Just moving out, maybe getting my own place. It's impossible to get a mortgage or a place like a house in this time, you know. But something that I can call my own, and you know, and just be financially stable and making sure my daughter is in school and having a good life. (*Isla*).

All the mothers wanted to leave social housing eventually but recognised this was unlikely without a stable, well-paid job.

Despite their negative experiences, some participants were resigned to remaining in social housing: ‘I never thought of actually leaving [social housing]. I just thought of staying and just moving elsewhere, because I've only heard bad things about certain buildings. There are a few buildings I've heard a lot of good things about.’ (*Alyssa*). Another ‘persistently unemployed’ participant was ‘improving’ her apartment by painting/renovating: ‘I figure I'm gonna be there probably, like potentially the rest of my life… Like it's horrible… it's just like I can't live in an eggshell-coloured world.’ (*Chloe*).

Some participants, particularly amongst the ‘persistently unemployed’ and ‘single mothers’, were reticent about working for fear of instability and being in a worse financial situation:

I've thought about [finding work]. I would like to, but I know it's a hard part with being on [social] housing and on ODSP, where you can only earn so much before they start to deduct stuff, and then my rent goes up, my ODSP goes down. And then, after so long I get taken off both of them. So, if I get taken off them, what happens if I can't keep that job and something happens? What do I fall back on then…? (*James*).

Similarly, some participants appeared to have disengaged from work and social connections to reduce vulnerability and risk, fearing losing their housing because of paid employment. Keeping to oneself was a shared theme, even for participants with more positive experiences of social housing. Withdrawing from other tenants appeared to be a protective mechanism, especially for participants in rougher housing areas, and social isolation also seemed to precipitate withdrawal from the quest for employment and other social engagement.

In contrast, other participants recognised the danger of a ‘welfare trap’ and sought to contradict the social housing stereotypes. An employed ‘single mother’ was trying to write herself a different employment story despite her history of growing up in social housing with a single mother and following a similar trajectory. She described the risks of relying exclusively on government benefits: ‘…like some people, it sounds really harsh, but when you're enabled, why would you want to go work? …if you're already getting your rent paid, and you know you're gonna get an income every month, there's no need to work.’ (*Isla*). Two ‘persistently unemployed’ participants actively challenged perceived social expectations by advocating for themselves, sometimes creating conflict with others. One participant explained that she was unable to return to formal employment due to her mental health problems, yet she remained innovative and creative, renovating and selling abandoned furniture. Similarly, one ‘youth’ described a future orientation that broke free of social expectations assigned to social housing residents:

I guess there's a stereotype that poor people or people who live in affordable housing are going to be more bummy. And I think [my girlfriend] and I, and there's a few other examples on this street that… go against that stereotype… like we both try, and we both have big aspirations… She doesn't want to be working minimum wage for the rest of life. Neither do I. And we both refuse to take any welfare, because the type of, like, mindset that we fear that would get us in like, 'Oh, we're making money so no point getting a job.' (*Lucas*).

#### Going it alone: Inadequate support

Lack of support compounded the precarity of employment and housing for participants. Nearly all participants shared feeling inadequately supported emotionally, physically, and materially from both formal and informal sources, either consistently or at some point in their lives. Some had received support initially, but then were left to manage ongoing, complex challenges alone, as this ‘persistently unemployed’ participant described: ‘After [formal community services] had gotten me on Disability [benefits] and found housing, it was basically they didn't have any more things that they could do for me’ (*James*). Several spoke about being compelled to ‘figure things out themselves’ because they perceived no one else cared or was available to support them e.g., ‘[Support services] never got me a job. Any job I’ve gotten was myself… I had to work for everything I had… I was making 600 a week at 12 years old… I was paying my mum rent.' (*Mark*).

Participants described lack of support in terms of skill development and work preparation, as well as encouragement to continue working. Many participants thought their education had not prepared them for work and few had received adequate training and professional development, often because they were in low-skilled, low-paid jobs. Concern that earning income while on government benefits would lead to financial precarity was raised, as one ‘single mother’ expressed: ‘I make too much for welfare… I'm on that line… but I make not enough, you know, to live on my own. Like it's just where you cross the line, I don't know.’ (*Isla*).

Additionally, relationship breakdowns reduced available support for participants to pursue employment. For example, one ‘youth’ lost his job because of conflict with his father: ‘I feel like that's what's happened at a lot of my jobs where something's going on at home, and I just I can't, I can't deal with work because of it.’ (*Ethan*). All the ‘single mothers’ had been through break-ups with partners, shouldering most of the care responsibilities for their children; this compounded their struggle to manage work or consider applying for jobs, even though some had access to valued family support: ‘My mother was a blessing, so she was a huge help for me, with childcare and stuff like that.’ (*Mia*).

Inadequate mental health support was another major barrier to employment, as one ‘persistently unemployed’ participant expressed: ‘Mental health needs to be way more looked after and taken care of, and people need to be listened to… that's the biggest issue that I've had with my mental health struggles and whatnot, is not being listened to…‘ (*Chloe*). One ‘single mother’ no longer received formal support despite struggling with depression and post-traumatic stress disorder from an abusive relationship. However, although she felt previous services were mostly ineffective, she expressed gratitude for a psychiatrist whose diagnosis helped her understand herself better: ‘…when I was diagnosed it was the first time that I ever started looking into it and realising oh, it's not me. There actually is something in my brain that's not functioning properly.’ (*Dakota*). Despite several ‘youth’ and younger ‘single mothers’ recognising their need to stabilise mentally before actively seeking employment, they had minimal engagement with formal support services. In contrast, though not currently working, one ‘youth’ expressed satisfaction with his current support: ‘My previous [support] worker still helps me from time to time, I think… I think I have some people I can ask for support.’ (*Nick*).

## Discussion

This study explored the employment experiences and goals of young adults living in social housing, and how this particular residential context intersects with employment. Through inductive analysis, three distinct narratives were identified — the ‘persistently unemployed’, ‘single mothers’, and ‘youth’ — broadly illustrating diverse employment trajectories. Despite their heterogeneity with respect to employment, participants’ shared context shaped their priorities, experiences, and goals.

Similar to other marginalised populations internationally,^46–48^ all participants described the importance of employment and both positive and negative past work experiences, but only two were employed at the time of interview and many described significant barriers and the need for more support. Employment was not viewed as a likely future option for the ‘persistently unemployed’. For ‘single mothers’, ensuring a better future for their children was a primary motivator for valuing work. The ‘youth’, at an earlier life stage, were considering employment options and generally more optimistic. Across groups, limited skills and education, work histories in inflexible low-paid and low-skilled work environments, compromised mental well-being, and inadequate supports—from childcare to formal employment services—were common. This discussion highlights the complex relationship between employment, housing, and well-being through a Capabilities lens, examining work and housing as opportunities shaped by enabling or constraining conversion factors.

### Social housing as both security and instability

Our findings highlight how social housing can provide both security *and* instability, affecting other capabilities and overall flourishing. Kimhur calls for considering ‘what conditions, abilities, opportunities or capacities a person needs for expanding her freedoms to choose a housing-relevant-functioning that she has reason to value (e.g., residing in a way that she values)’.^41 p268^ In the context of Canada's housing crisis, participants expressed gratitude for affordable housing, and many had experienced long waitlists. Given the ‘complex web of requirements and documentation’^22 p219^ required to secure affordable housing, residents may avoid employment if it is perceived to threaten housing security,^49^ particularly where housing supply is limited and commercial rents are high. Hence, while social housing provides essential shelter, it does not necessarily enable other valued capabilities such as accessing, sustaining or progressing in meaningful employment.

Beyond income poverty, various social and environmental conversion factors create instability and constrained capabilities. Participants in older buildings described neglected, unsafe conditions (e.g., pests, drug use, fear of victimisation) that hindered their capability to gain and sustain employment (e.g., cross contaminating a workplace with pests) and maintain well-being. Housing location and limited transport were additional environmental barriers for some participants. Stigma and safety concerns increased participants’ disengagement and isolation, reducing access to support and community participation—conditions linked to unemployment.^16^ Concentrated disadvantage and the reputation of certain buildings further restricted employment opportunities. Thus, many participants appeared to live in a double bind, tolerating social and environmental challenges to ensure some housing stability.

Additionally, housing and welfare policies related to income thresholds (social and environmental conversion factors), as well as limited knowledge and misconceptions about earning thresholds (personal conversion factors) also constrained employment capabilities. Fear that employment income would jeopardise housing or other supports, particularly if they were responsible for children or believed that maintaining a job was tenuous based on their work histories, made disengagement from formal work a reasonable protective response.^48^ Peter and Polgar^50^ critique the ‘welfare-to-work paradox’ wherein Canadian welfare recipients are pressured into low-paying, insecure work which then decreases their social assistance, increases costs, and requires constant income reporting — demoralising and disincentivising work participation. Conversely, several participants in our study perceived a potential ‘welfare trap’ in social housing that discouraged employment and negatively shaped priorities, choices and capabilities.

Our findings highlight the pervasive, complex, and vicious cycle between mental health, social housing, and employment. Although formal diagnoses were not obtained, compromised mental health, another personal conversion factor, was prevalent, and participants described anxiety, depression, fear, self-doubt, and inertia. Mental health issues also presented in alternate forms, including safety concerns, expectations of stigma, messages from others suggesting lack of capability, isolation, limited work skills and experiences, conflictual family relationships, and sole parenting pressures. Poor mental health is linked to job loss and precarious work,^17,18,31^ while repeated failure to secure employment can lower self-esteem and reduce motivation and hope, particularly for young adults with minimal qualifications.^16^ Beyond the individual, precarious employment can affect households, delaying life-course events (e.g., marriage, children) and increasing tensions and feelings of inferiority.^51^ Additionally, precarious employment can directly and indirectly — through precarious housing — contribute to chronic stress, demonstrating the combined burden of precarious employment and housing on mental health.^52^

Likewise, research indicates that living in social housing can exacerbate mental health challenges^53^ with a cumulative negative effect on mental health, particularly with multiple transitions.^54,55^ Environmental factors related to poor housing conditions, including maintenance delays, surrounding garbage, pests, exposure to crime, violence and drug-use, and social factors such as low social cohesion, lack of positive role models and social support, can all contribute to poor mental health.^53,56,57^ While a recent Canadian study found that mental health improved significantly after 12 months for social housing recipients compared to others on waitlists,^58^ the authors postulated this was likely due to decreased uncertainty and financial stress and improved housing conditions. Hence, the effect on mental health may be more dependent on specific characteristics of social housing environments, including housing quality and social cohesion,^57^ which can either support or hinder capabilities.

### Capabilities and reclaiming employment

Engaging in work is a valued undertaking for Canadian young adults, particularly when characterised by fairness, equity, and dignity. As a multi-dimensional framework, the Capabilities Approach considers work as contributing to valued capabilities and overall flourishing beyond productivity or income^41,59,60^; hence, it can be useful for identifying what opportunities and freedoms (capabilities) social housing residents have or need to realise the multiple well-being and inclusion benefits of employment.

Current employment services do not necessarily promote capabilities, often prioritising rapid job entry (‘work-first’) that can disregard choice, safety or work's value/meaning — ‘one-size-fits-none’ policies and programs.^16,61–63^ Even charitable employment programs are constrained within inequitable systems and may offer only limited/obligatory choices.^29^ Services that restrict choice and disregard participation in other meaningful occupations (e.g., self-care, civic participation, leisure), often perpetuate cycles of precarious work, unemployment, and poor health.^50^ Additionally, vocational rehabilitation programs may fail to offer holistic support, including addressing pervasive interpersonal challenges.^64^ Employment quality, not simply employment status, affects health, well-being and productivity, yet unemployment can constrain opportunities, reduce prospects and push people to accept poorer quality jobs.^65^

Muñoz and Rosario^66^ advocate moving beyond human capital approaches where individuals are a means to an economic end, towards capability-focused approaches that support human development and recognise work as one aspect of well-being. For example, Payne and Butler^67^ studied a UK-based third-sector employment program that provides personalised support to individuals with complex needs to determine the influence of choice on well-being. Despite many individual and environmental barriers to work and low expectations, users felt supported by the program to make choices, expand their horizons, and consider whether they were better off working or not; this was facilitated through genuine, respectful, trusting relationships with program workers and holistic support (e.g., childcare, transport subsidies, facilitating social interactions, navigating systems). Similarly, Pearson et al.^59^ highlight the importance of ‘relational employability’ in supporting Scottish single parents to work; collaboration and mutually respectful relationships between program workers and users were crucial for supporting choice in valued work.

### Policy and practice implications

Capabilities-aligned policies and supports could help address the substantive personal, social and environmental barriers faced by young adult social housing residents. Firstly, financial investment in social housing could contribute to more positive environments as a foundation for supporting young adults’ capabilities and participation in life roles, including employment. During the 1990s with increasing neoliberalisation, investment in Canadian social housing was significantly cut and it remains severely underfunded despite modest improvements in the early twenty-first century.^68^ A recent Canadian bank report calls for doubling of social housing stock as a first step in supporting marginalised Canadians.^69^

As well as increasing social housing stock, investment in building neighbourhood pride and community engagement could improve mental health, encourage active citizenship, and create opportunities for skill development, income generation, and employment networks. Initiatives such as green spaces or community gardens within social housing can build knowledge and connection to nature, enhance safety, belonging, and community pride.^70–72^ Additionally, timely maintenance could support residents feeling valued and improve safety and well-being, as inadequate/delayed maintenance and pest problems can exacerbate poor health, powerlessness and anxiety.^2,56,72^ Such interventions would enhance the opportunity structure and improve employment capabilities for social housing residents.

Existing employment support models developed for marginalised populations also hold potential if adapted for the specific needs of young adults in social housing. The Individual Placement and Support (IPS) model has been implemented and evaluated extensively in community mental health to help people experiencing serious mental illness find and keep personally meaningful work. This intervention, whose core elements have been well described and researched, includes individual collaboration and support to identify interests and secure and maintain work in mainstream employment, meeting the range of needs and challenges faced by job seekers.^73^ IPS is considered a best practice in community mental health and research has demonstrated its effectiveness across populations, including for individuals with anxiety, depression, posttraumatic stress disorders, substance use disorders and intellectual disorders.^73^ Further investment in this approach and connecting social housing providers (e.g., social housing support managers) with local IPS providers could, therefore, support capabilities for social housing tenants seeking employment.

Another strategic approach consistent with a Capabilities Approach is work integration social enterprise (WISE). Like all social enterprise, WISEs are businesses with both commercial and social missions, providing goods and services within a community marketplace to achieve their social goal of improving employment and social integration outcomes among individuals marginalised from the broader workforce.^74,75^ A growing body of research demonstrates the health, well-being and employment-related outcomes of WISE, along with important organisational features, e.g., for socially/economically disadvantaged young people^76^ and homeless youth.^77^ A WISE might, for example, bring young adult social housing residents together to envision a business opportunity based on their interests and community needs, collaborate with business experts to plan, develop and register a business, and evolve business structures and practices sensitive to their needs for support and skill development [e.g.,^78^]. However, successful implementation requires deep contextual knowledge, including for decisions around work activities, hiring processes, temporary versus permanent employment, wages, and appropriate accommodations such as flexibility, security and supports.^79^ Again, such an endeavour requires partnerships between social housing providers and local businesses and community services, which could be facilitated through a research collaboration such as the current study.

In alignment with the Capabilities Approach, our findings suggest that providing social housing as a resource alone is insufficient for young adults to engage in preparing for, seeking, and sustaining employment. Firstly, any support approach must integrate accessible, current information about earning thresholds and government benefit requirements to ensure informed decision-making around employment. Secondly, practical support such as transport subsidies and affordable, flexible and accessible childcare is essential. Thirdly, integration of opportunities for further education, training and mentorship are important. Employment support interventions that are sensitive to the link between limited educational and work histories, neighbourhood social and economic resources, and material deprivation may effectively enhance the likelihood of obtaining secure employment.^35^ Fourthly, many participants described mental health challenges with little to no formal support, reflecting the limited access to mental health services for marginalised Canadian young adults^80^ and the prevalent ‘lack of collaboration between the housing and the health sectors and a general lack of information concerning available services in [social housing residents’] residential environment.’^72 p298^ Integration of mental health-related support with employment services as well as relationships with mental health services are necessary^47^ and are established features of evidence-based IPS and WISE. Considering the current fragmentation of support services, cross-sector partnerships between housing, health, and employment sectors are critical.

### Limitations

Several limitations to this research should be considered when interpreting the findings. Despite significant recruitment efforts, our sample size remained small and focused on a specific social housing corporation in a particular mid-sized Canadian city; therefore, as for all qualitative research, these findings will not reflect all social housing populations and employment contexts. Nevertheless, our findings can provide insights that are potentially transferable to similar contexts, particularly in similar-sized Canadian cities and with social housing corporations that function primarily as landlords rather than providing support services. Due to the small sample size, some perspectives were likely underrepresented, and an intersectional analysis was not possible; however, exploring how various forms of marginalisation (e.g., poverty, race, gender) and lack of generalised well-being affect employment participation would be an important area of future research. The study recruitment script was framed around employment and, despite reassuring participants that interviews were confidential and identifying information would be removed, fears of sharing work-related information not disclosed to the housing corporation or stigma around unemployment may have reduced participation or disclosure. Additionally, health conditions were self-reported and may not accurately or comprehensively encompass the range of mental and physical barriers experienced by participants. All interviews were conducted using an online platform which could have hindered rapport; however, participants also expressed appreciation for the convenience and possible anonymity (i.e., choosing to keep their video off). Finally, this study was cross-sectional and therefore we were unable to explore how employment experiences, needs, challenges and goals may change over time for individuals—an important avenue for future research.

## Conclusion

This study explored the employment experiences, priorities and goals of young adult social housing residents as a foundation for further research to address challenges and implement appropriate supports. Participants faced numerous barriers to navigating the double precarity of vulnerable housing and insecure employment or unemployment. Despite only two participants being formally employed at the time of interview and many describing past experiences of precarious work, most valued employment but lacked the necessary support, opportunities and choices needed for meaningful work. Participants described how the social housing context both facilitated and hindered employment. Consistent with the Capabilities Approach, resources must be combined with holistic support to enable real opportunities for employment and other valued occupations.^60^ This study highlights the complexity of encouraging sustainable labour-force participation for young adult social housing residents, suggesting implications and several conditions required as part of a comprehensive yet individualised employment strategy to promote overall flourishing.
